# Surveillance of respiratory viruses among children attending a primary school in rural coastal Kenya

**DOI:** 10.12688/wellcomeopenres.15703.2

**Published:** 2020-09-24

**Authors:** Irene Wangwa Adema, Everlyn Kamau, Joyce Uchi Nyiro, Grieven P. Otieno, Clement Lewa, Patrick K. Munywoki, D. James Nokes

**Affiliations:** 1 Epidemiology and Demography Department, KEMRI-Wellcome Trust Research Programme, Kilifi, 80108, Kenya; 2 School of Life Sciences and Zeeman Institute for Systems Biology and Infectious Disease Epidemiology Research (SBIDER), University of Warwick, Coventry, Coventry, CV4 7AL, UK

**Keywords:** Respiratory viruses, acute respiratory infections, school surveillance, real-time PCR, school children, nasopharyngeal samples, coastal Kenya

## Abstract

**Background: **Respiratory viruses are primary agents of respiratory tract diseases. Knowledge on the types and frequency of respiratory viruses affecting school-children is important in determining the role of schools in transmission in the community and identifying targets for interventions.

**Methods: **We conducted a one-year (term-time) surveillance of respiratory viruses in a rural primary school in Kilifi County, coastal Kenya between May 2017 and April 2018. A sample of 60 students with symptoms of ARI were targeted for nasopharyngeal swab (NPS) collection weekly.  Swabs were screened for 15 respiratory virus targets using real time PCR diagnostics. Data from respiratory virus surveillance at the local primary healthcare facility was used for comparison.

**Results: **Overall, 469 students aged 2-19 years were followed up for 220 days. A total of 1726 samples were collected from 325 symptomatic students; median age of 7 years (IQR 5-11). At least one virus target was detected in 384 (22%) of the samples with a frequency of 288 (16.7%) for rhinovirus, 47 (2.7%) parainfluenza virus, 35 (2.0%) coronavirus, 15 (0.9%) adenovirus, 11 (0.6%) respiratory syncytial virus (RSV) and 5 (0.3%) influenza virus.  The proportion of virus positive samples was higher among lower grades compared to upper grades (25.9% vs 17.5% respectively; χ ^2^ = 17.2, *P* -value <0.001). Individual virus target frequencies did not differ by age, sex, grade, school term or class size. Rhinovirus was predominant in both the school and outpatient setting.

**Conclusion: **Multiple respiratory viruses circulated in this rural school population.  Rhinovirus was dominant in both the school and outpatient setting and RSV was of notably low frequency in the school. The role of school children in transmitting viruses to the household setting is still unclear and further studies linking molecular data to contact patterns between the school children and their households are required.

## Introduction

Acute respiratory infections (ARI) pose a globally important disease burden and are a major contributor both to morbidity and mortality notably attributed to pneumonia ^1, 2^.

Bacterial pathogens are important disease agents of the respiratory tract. However, considerable efforts have been made in the fight against bacterial causes of ARIs. Interventions such as conjugate vaccines ^3– 5^ are changing focus of the etiology of ARIs to respiratory viruses ^6^. A recent multi-country severe pneumonia etiology study in sub-Saharan Africa and South Asia reported 61% of hospitalized cases were caused by viral pathogens and 27% by bacterial agents ^7^.

Respiratory viruses have been identified as the primary agents of mild disease of the upper respiratory tract ^8– 10^. Studies conducted in a number of African countries consistently show that one or more respiratory viruses are responsible for the majority of the ARI cases reported yearly ^1, 8, 11– 15^. ARIs in school children are most times mild and affect the upper respiratory tract often presenting as the common cold, a self-limiting viral infection involving the nose, sinuses, pharynx and larynx ^16^. A small proportion will develop more severe illness ^17^. In addition to the direct medical costs incurred due to the burden of medically-attended respiratory illnesses, ARIs in young school age children have far reaching effects which include missing out on school days for sick children and missed work days for parents who have to take care of ill children ^16^.

Options for prevention and control of respiratory viruses are limited. No vaccines are presently available for the main respiratory viruses, with the notable exception of influenza. In the absence of viral vaccines, design of the effective control measures now pivots on understanding the mechanisms of spread of these viruses in different settings. In studies analyzing the effectiveness of intervention strategies for ARIs the assumption is that children play a central role in respiratory virus transmission in the community ^18^. Prevention or reduction of occurrence of ARIs in school-going siblings is likely to result in a reduction of infections to the vulnerable infants in the households ^19^ and to the elderly in the community ^20, 21^ Knowledge on the different types of respiratory viruses affecting school children and factors associated with ARI in this setting can further aid in identifying modifiable factors which are viable targets for interventions.

The present study forms part of a larger project titled SPReD ( Studies of the Pathways of transmission of Respiratory virus Disease) which aims to advance understanding of the nature of spread of respiratory viruses (e.g. RSV, influenza, coronavirus, rhinovirus) at different scales of observation from the individual, household and school, local community to countrywide, and use this information to innovate interventions. This work presents results from a one-year surveillance of respiratory viruses in a rural primary school in Kilifi county, coastal Kenya.

## Methods

### Study design

This is a longitudinal study collecting and analyzing data from an open cohort of 469 students. Participants were followed up for a period of one school year from May 2017 to April 2018, equivalent to three school terms; vacation periods were not included.

### Study site

The study was undertaken in a school in Kilifi County, coastal part of Kenya, a rural area 3° south of the equator, off the Indian Ocean coast, typical of much of tropical sub Saharan Africa. The study site was purposively selected from a list of nine locations within the Kilifi Health and Demographic Surveillance System (KHDSS) participating in a study on the transmission pathways of viral respiratory infections in Kilifi County ^22^. The KHDSS area was established by the KEMRI Wellcome Trust Research Programme (KWTRP) in 2000 ^23^ to monitor births, deaths, in-migration and out-migration in a population of approximately 296,000 residents (2016 census-unpublished data, data is available on request from KEMRI) over an area of 891km ^2^.

Junju location, a rural, low mobility and low socio-economic status setting, with 13 primary schools, was purposively selected because of the long-standing relationship and trust between its residents and its local health facility and the research institute, KWTRP. The proposed intensive study, targeting children, a vulnerable population, with weekly school visits for sampling, required careful selection, sensitization and prior engagement to realize acceptance and a high response rate. The selected primary school had daycare, pre-primary and primary school children of both genders. The school children could access a health facility, where surveillance of respiratory viruses was ongoing ^22^. Contemporaneous surveillance of childhood pneumonia admissions to the Kilifi County Hospital was also ongoing. These were important for comparison of viruses circulating in the school with those from the community reported at the health facility, and admissions to the local referral hospital.

### Enrollment, sampling and sample collection

Before commencement of the study, over a period of three months, engagement meetings were held with the Kenya Ministry of Health and Ministry of Education officials to acquire permission to conduct the study among primary school children. Sensitization meetings were held with the School Board, the teachers, the Parent Teacher Associations (PTA) and the parents. Open day sessions were held at the school to sensitize the students on scientific topics, the objectives of the study and procedures involved. Ethical approval was sought from the KEMRI Scientific and Ethics Review Unit (SERU).

An open cohort design was adopted where new admissions to the school and students not initially enrolled to the study were permitted to join in terms two and three. After successful community engagement activities, students from the 12 grades in the school and their teachers were enrolled into the study. Students were divided into two main groups, the lower primary and the upper primary. Day care, kindergarten 1 to 3 and grade 1 (age range 2 – 12 years) were classified as lower primary and grades 2–8 (age range 6 – 19 years) classified as upper primary. Demographic details (age, sex, grade, height, weight, mid upper arm circumference) of all students who gave consent were collected.

### Sampling

Data on ARI symptoms were captured daily in seven-day symptoms diaries labelled which were administered at the beginning of each week and filled out every day of the week including weekends by each participant in upper primary (grade 2 and above). Students in the lower grades (day care to grade 1) were too young to fill out the registers. Field staff assisted by assessing the students in the lower grades every weekday for ARI symptoms and filled in the symptom diaries on their behalf. On the day of sample collection, students with ARI symptoms were identified by checking their flu diaries (Analysis of surveillance of symptomatic ARI is presented in a separate manuscript). For each of 42 school-weeks, a convenience sample of 60 students with more than one ARI symptom of either cough, nasal discharge or sore throat on the day of sample collection was targeted for nasopharyngeal swab (NPS) collection at the school (total sample of 2520 targeted over school year). This represented 8 samples per grade per week from the lower primary (5 grades) and 4 samples per grade per week from the upper primary (7 grades). Oversampling among the lower grades was motivated by the perceived critical role of this age group for childhood infectious diseases and hence the need to reduce the level of uncertainty in the estimated risk for this age group (for example 22, 24). Based on the range of prevalence for respiratory viruses in ARI presentations from a study in health facilities in the KHDSS ^22^, this sample size was estimated to yield a prevalence range of 7%, 95%CI (6.1- 8.1) to 15%, 95%CI (13.6-16.5).

NPS samples were collected by trained field workers. For pragmatic reasons, sampling was usually initiated on Wednesday of each week. A count of all students presenting with more than one ARI symptom was first conducted from which the required sample was selected. On the days when the symptomatic students were less than the required number of samples per grade, per week, all symptomatic students gave a sample. The procedure was repeated the following day until the required number of weekly samples was obtained. If the symptomatic students were more than the maximum number required, randomization was done. This involved using several cards equivalent to the number of symptomatic students on that day. Some cards were marked “give sample” and others “no sample”. Data and sample collections were not conducted when the schools were on vacation. A pilot study was conducted in March 2017 for a period of one month. Samples collected during this phase were excluded from this report.

### NPS sample and data collection

NPS samples were collected using the standard procedure for deep nasopharyngeal specimens previously described ^25^. Briefly, a flocked swab (503CS01, Copan Diagnostics, Flocked Swab Technologies, Italy) is inserted into one nostril as far as the deep nasopharynx, gently twisted three times before slowly withdrawn ^26^ (10 seconds procedure). NPS swabs were transferred to a single 1ml vial of viral transport media (provided by COPAN Diagnostics, catalogue number 331c) and stored in a cool box kept at ~2-8 °C before transportation to KWTRP labs within 4 hours after sample collection. Data on symptoms experienced and recent travel were collected using short questionnaires on computer tablets and entered in real time (see extended data ^27^).

The recruitment and specimen collection procedures in the school during the first school term of 2018 coincided with recruitment and specimen collection at the local Junju dispensary (primary health care facility) which was part of the KHDSS-wide surveillance as described elsewhere ^22^. Patients of any age presenting with one or more ARI symptoms of cough, sneezing, nasal congestion, difficulty breathing, or increased respiratory rate for age were eligible. NPS collection was integrated into the routine procedures. Samples from the clinic were collected twice weekly with a maximum of 15 samples per week (details on the sample size and sampling procedures are detailed in the referenced manuscript) ^22^. Children under 60 months of age admitted to the Kilifi County Hospital with syndromic severe or very severe pneumonia were enrolled as part of a continuous respiratory virus surveillance study, with NPS samples collected (details published elsewhere ^28^). Only children considered to be appropriately aged for their school grade are included in this analysis. Those >10 years in lower primary were considered too old for their grade, while those aged <10 years and in upper primary were considered too young for their grade. Results from NPS samples obtained from those children, as well as the results of samples obtained from school teachers, will be presented in separate manuscripts.

### Laboratory procedures

At the KWTRP labs samples were aliquoted into 2 vials each containing 0.5ml and stored at -80°C awaiting processing. Ribonucleic acid (RNA) was extracted from the NPS specimens and screened by molecular diagnostic assay, using previously described methods ^22^. In summary, RNA was extracted using RNeasy extraction QIA-cube HT kit (Qiagen, Germany, catalogue number 74171) from 140μl of the swab sample. This was performed according to manufacturer’s instruction except that carrier RNA was added to the protocol and eluted to 100μL instead of 200μL. Extracted samples were screened for a range of 15 virus targets; RSV (A and B), human rhinovirus (HRV), human coronaviruses (HCoV OC43, NL63, E229), influenza viruses (Flu- A, B, and C), parainfluenza viruses (PIV 1-4), adenovirus (ADV) and human metapneumovirus (HMPV); using a multiplex (MPX) 7500 real-time PCR assay system from Applied Biosystems. Cycling parameters used were 50°C for 20 minutes, 95°C for 5 minutes, 40 cycles of 95°C for 15 seconds and 40 cycles of 60°C for 30 seconds. We assumed the virus load to be inversely related to the cycle threshold (Ct) value for each test sample. No housekeeping gene were co-analyzed. However, included control samples (RNA or PCR products) for each target group in each 96 well plate. The MPX assay included *Mycoplasma pneumoniae*, but it was not considered in this report.

### Statistical analysis

Data were cleaned and statistical analysis conducted using STATA 14.1 (College Station, Texas). Descriptive analysis such as frequencies, proportions and percentages were performed to generate baseline characteristics of the participants. Summaries of virus detections by person, place and time characteristics were generated after accounting for the sampling design by applying sampling weights. Age dependent mid upper arm circumference (MUAC) measures ^29^ were used to estimate undernutrition. This was based on the validated MUAC-for-age z-score growth curves by sex for children aged 5 – 19 years. This MUAC for age growth reference accords with WHO 2005 growth standards ^30^.

Sampling weights for the lower and upper classes were calculated separately as the inverse of the probability of selecting a student to give a sample in the lower grades and upper grades (inverse of the total number of samples to be collected divided by the total number of students in the stratum). The weights were then assigned to each student according to their grade level. Weighted estimates were obtained using the “svy” commands in Stata.

Graphs of temporal patterns of virus detections by month and school term were produced. The Chi-Square test of association was used to test the association between infection and the risk factors.

### Ethical considerations

The study was approved by the Kenya Medical Research Institute-Scientific Ethics Review Unit (KEMRI-SERU #3332) and the University of Warwick Biomedical and Scientific Research Ethics Committee (BSREC #REGO_2016-1858). Written informed consent to participate in the study was obtained from the relevant school authorities, and all parents and guardians of the students who participated in the study. Assent was obtained from all students above 13 years of age, following set guidelines for informed consent process when involving teenagers or minor adults in research conducted at the Kenyan coast ^31, 32^. Any child aged 13 years or less who explicitly refused to participate was excluded.

## Results

### Baseline characteristics

Out of a total 781 students distributed in 12 grades (daycare, Kindergarten 1 – 3 and grade 1 – 8), 469 students (60%), aged 2–19 years who had parental and student consent were enrolled into the cohort. Students were followed up for three school terms between May 2017 and April 2018 (220 school days). 

Of those enrolled, 253 (54%) were female. Grade 1 had the highest number of participants (72, 15.4%), while kindergarten year 1 and 3 had the lowest, (19, 4.1%). Students in the lower primary had a median age of 6 years (range; 2 – 9) and those in upper primary a median age of 12 years (range; 9 – 19) at enrollment. The median number of students per class was 66 (range; 30 – 95); 371 students (79.1%) had a low MUAC for age score, an indication of undernutrition. Table 1 provides the baseline characteristics of the enrolled students (See underlying data for full study data ^27^).

**Table 1.** Baseline characteristics of participants in a rural primary school in coastal Kenya, year 2017–18.

Characteristic	Number of Participants (%) # N= 469	Number of students Sampled (%) # n=325	Number of Samples (%) # n = 1726 **
**Age category (yrs.)**			
**2–5**	22 (4.7)	21 (6.5)	127 (7.4)
**5–9**	137 (29.2)	95 (29.2)	855 (49.5)
**10–14**	224 (47.8)	151 (46.5)	538 (31.2)
**15 –20**	86 (18.3)	70 (21.5)	206 (11.9)
**Sex**			
**Male**	216 (46.1)	152 (46.8)	834 (48.3)
**Female**	253 (53.9)	173 (53.2)	892 (51.7)
**Class Level**			
**Lower Primary**	167 (35.6)	113 (34.8)	982 (56.8)
**Upper Primary**	302 (64.4)	212 (65.2)	744 (43.1)
**Grade**			
**Daycare**	24 (5.1)	23 (7.1)	178 (10.3)
**Kindergarten1**	19 (4.1)	16(4.9)	178 (10.3)
**Kindergarten 2**	33 (7.0)	28 (7.8)	252 (14.6)
**Kindergarten 3**	19 (4.1)	14 (4.3)	233 (13.5)
**Grade 1**	72 (15.4)	32 (9.8)	141 (8.2)
**Grade 2**	47 (10.0)	30 (9.2)	88 (5.1)
**Grade 3**	53 (11.3)	36 (11.1)	107 (6.2)
**Grade 4**	40 (8.5)	31 (9.5)	127 (7.3)
**Grade 5**	43 (9.2)	37 (11.4)	128 (7.4)
**Grade 6**	34 (7.3)	20 (6.1)	110 (6.4)
**Grade 7**	47 (10.0)	29 (8.9)	106 (6.1)
**Grade 8**	38 (8.1)	29 (8.9)	78 (4.5)
**School Term**			
**2017 Term 2**	404 (85.7)	266 (81.8)	767 (44.4)
**2017 Term 3**	372 (79.3)	191 (58.7)	417 (24.1)
**2018 Term 1**	336 (71.6)	176 (54.1)	542 (31.4)
**Symptoms**			
**Cough**	365 (77.8)	272 (83.7)	1280 (74.1)
**Runny Nose**	379 (80.8)	305 (93.8)	1511 (87.5)
**Sore Throat**	175 (37.3)	140 (43.1)	350 (20.3)
Class Size *			
**<=45 Students**	62 (13.2)	53 (16.3)	589 (34.1)
> **45 Students**	407 (86.8)	272 (83.7)	1137 (65.8)

^#^ Shows percentage in category of characteristic *class size 45= recommended class size ** Excludes 10 samples that were not analyzed

In total, 429/469 students (91.5%), experienced acute respiratory illness symptoms at least once during the study period. Only 413/469 (88.1% ) of the students had a combination of more than one symptom during the study period, and qualified for NPS sampling. NPS samples were collected from 325/420 students, constituting 77.4% of those who experienced ARI symptoms during the study period ( Table 1). Overall, 1726 samples were collected. There were 78 students who were sampled once, 59 sampled twice, 39 sampled thrice, 29 sampled four times, 20 sampled 5 times, and 100 students were sampled more than five times. The highest number of samples collected from a student was 30. Due to the sampling regime, samples from students between 5–9 years constituted approximately half of all samples collected (49.5%, 855/1726 samples), whereas samples from students below 5 years contributed only 7.4% of samples collected. The most common symptom among sampled students was nasal discharge and the most common symptom combination was cough and a nasal discharge (72%). Distribution of students who gave NPS samples and number of samples collected by demographic characteristics is shown in Table 1.

### Virus detections from NPS samples

A total of 1726 samples were collected from 325 students. Samples at least positive for one virus were 384 (22.2%). Out of the students sampled, 176 (54.2%) had at least one virus detected. Of students with virus positive samples, 59.3% had a positive detection only once during the study period. Multiple infections were detected in 79 students, constituting 24.3% of the students sampled, and 44.9% of students with virus positive samples. The highest number of single infections detected was 12 out of 21 samples collected from one student in lower primary aged 5 years. (8 rhinovirus, 1 adenovirus, 1 influenza type B, 1 parainfluenza type 3 and 1 parainfluenza type 4). 

The median age of the students with virus positive samples at the time of sample collection was 8 years (IQR, 6–12). The proportions of virus positive samples differed significantly by age, sex, grade level, school term and classroom size after applying sampling weights ( Table 2). The proportion of virus positive samples was higher among lower primary students (daycare to grade 1) compared to upper primary students (25.9% vs 17.5% respectively; *X* ^2^= 17.2, *P*<0.001). Similarly, male students had a higher proportion of virus positive swabs compared to female students despite contributing fewer samples; 25.4% vs 19.3% respectively; X ^2^ = 9.4; *P* = 0.037.

**Table 2.** Number and proportion of samples virus positive by participant characteristics, in a primary school in coastal Kenya, year 2017–18.

Characteristic		Any virus positive		Virus negative		P value
**Age category**	N	n	%	n	%	
**2–4**	127	38	29.9	89	70.1	
**5–9**	855	216	25.3	639	74.7	
**10–14**	538	93	17.3	445	82.7	
**15–20**	206	37	18.0	169	82.0	<0.003 *
**Sex**						
**Male**	834	212	25.4	622	74.6	
**Female**	892	172	19.3	720	80.7	0.037 *
**Class level**						
**Lower primary**	982	254	25.9	728	74.1	
**Upper primary**	744	130	17.5	614	82.5	<0.001 *
**Grade**						
**Daycare**	178	65	36.5	113	63.5	
**Kindergarten1**	>178	43	24.1	135	75.8	
**Kindergarten2**	252	58	23.0	194	77.0	
**Kindergarten3**	233	53	22.8	180	77.2	
**Grade 1**	141	35	24.8	106	75.2	
**Grade 2**	88	15	17.1	73	83.0	
**Grade 3**	107	30	28.0	77	72.0	
**Grade 4**	127	23	18.1	104	81.9	
**Grade 5**	128	21	16.4	107	83.6	
**Grade 6**	110	13	11.8	97	88.2	
**Grade 7**	106	18	17.0	88	83.0	
**Grade 8**	78	10	12.8	68	87.2	0.001 *
**School term**						
**2017 term 2**	767	134	17.5	633	82.5	
**2017 term 3**	417	102	24.5	315	75.5	
**2018 term 1**	542	148	27.3	394	72.7	<0.001 *
**Symptoms**						
**Cough**	1280	287	22.4	993	77.6	0.385 *
**Runny nose**	1511	346	22.9	1165	77.1	0.370 *
**Sore throat**	350	75	21.4	275	78.6	0.535 *
**Class size**						
**<=45 students**	589	161	27.3	428	72.7	
> **45 students**	1137	223	19.6	914	80.4	<0.001 *
*BMI						
**Underweight**	343	81	23.6	262	76.4	
**Normal**	1336	297	22.2	1039	77.8	
**Overweight**	47	6	12.8	41	87.2	0.156 *

* Weighted estimate *BMI- Body Mass Index

Students aged 2 - ≤5years made up 4.7% of the participants contributing to 7.4% of collected samples. Close to a third of these samples ((29.9%, 38/127)]) had one or more respiratory viruses detected. The proportion of virus positive samples was highest among students 2 - ≤5 years; χ ^2^ = 19.4; *P* <0.002. There was no statistical difference in the proportions of virus positive samples stratified by any of the symptom combinations, cough and nasal discharge, cough and sore throat or nasal discharge and sore throat.

**Figure 1.**
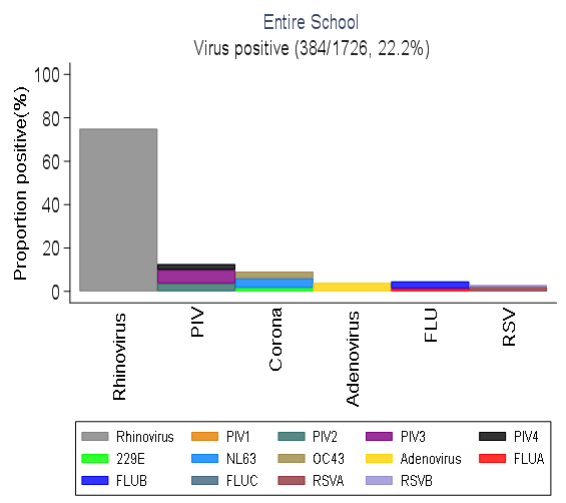
The distribution of six virus types detected in all samples collected in the entire school over the study duration. (RSV- respiratory Syncytial Virus; FLU- Influenza virus A, B, C; PIV -Parainfluenza virus 1-4; Corona- Human coronavirus NL63, E229, OC43; Rhinovirus-Human rhinovirus).

Of the 1726 samples screened, 13 out of 15 virus targets were detected (86.7%); no sample was positive from HMPV or parainfluenza virus type 1. These were collapsed into six respiratory virus groups. The frequency of virus positive samples by virus group was 288 (16.7%) for rhinovirus (HRV), 47 (2.7%) for parainfluenza virus, 35 (2.0%) for Human coronavirus (HCoV), 15 (0.9%) for adenovirus, 11 (0.6%) for RSV and 5 (0.3%) for influenza virus ( Figure 1). Rhinovirus was the dominant circulating virus detected in samples from nearly half of the students sampled (45.2%) and constituting 75% of the virus positive samples, as shown in Table 3. Proportions of RSV and coronavirus detections differed by school term (P =0.033 and P <0.001 respectively), virus targets did not differ by age, sex, grade level, school term or class size.

**Table 3.** Proportions of viruses detected by various characteristics from a rural primary school in coastal Kenya, year 2017–18.

Characteristic	Any virus n=384	RSV n=11	Adeno n=15	Rhinovirus n=288	PIV n=47	Corona n=35	FLU n=5
**Age category**							
**<5**	38	0 (0.0)	1 (2.6)	31 (81.6)	7 (18.4)	2 (5.3)	0 (0.0)
**5–9**	216	5 (2.3)	8 (3.7)	164 (75.9)	27 (12.5)	18 (8.3)	2 (0.9)
**10–14**	93	4 (4.3)	5 (5.4)	70 (75.3)	9 (9.7)	6 (4.5)	2 (2.1)
**>=15**	37	2 (5.4)	1 (2.7)	23 (62.2)	4 (10.8)	9 (24.3)	1 (2.7)
**Sex**							
**Male**	212	4 (1.9)	10 (4.7)	163 (76.9)	25 (11.8)	19 (9.0)	3 (1.7)
**Female**	172	7 (4.1)	5 (2.9)	125 (72.3)	22 (12.8)	16 (9.3)	2 (0.9)
**Class level**							
**Lower primary**	254	5 (2.0)	9 (3.5)	195 (76.7)	34 (13.4)	20 (7.9)	2 (0.7)
**Upper primary**	130	6 (4.6)	6 (4.6)	93 (71.5)	13 (10.0)	15 (11.5)	3 (2.3)
**Grade**							
**Daycare**	65	3 (4.6)	2 (3.1)	51 (78.5)	9 (13.8)	5 (7.7)	0 (0.0)
**Kindergarten1**	43	0 (0.0)	2 (4.7)	32 (74.4)	8 (18.6)	1 (2.3)	2 (2.3)
**Kindergarten2**	58	1 (1.7)	1 (1.7)	49 (84.5)	5 (8.6)	4 (6.9)	0 (0.0)
**Kindergarten3**	53	0 (0.0)	1 (1.9)	39 (73.6)	7 (13.2)	8 (15.1)	1 (1.9)
**Grade 1**	35	1 (2.9)	3 (8.6)	24 (68.6)	5 (14.3)	2 (5.7)	0 (0.0)
**Grade 2**	15	1 (6.7)	0 (0.0)	10 (66.7)	2 (13.3)	2 (13.4)	0 (0.0)
**Grade 3**	30	1 (3.3)	1 (3.3)	22 (73.3)	5 (16.6)	2 (6.7)	0 (0.0)
**Grade 4**	23	0 (0.0)	3 (13.0)	18 (78.3)	1 (4.4)	3 (13.0)	0 (0.0)
**Grade 5**	21	3 (14.3)	0 (0.0)	15 (71.4)	1 (4.8)	1 (4.7)	1 (4.8)
**Grade 6**	13	0 (0.0)	1 (7.7)	8 (61.5)	1 (7.7)	2 (15.4)	2 (15.4)
**Grade 7**	18	1 (5.6)	0 (0.0)	11 (61.1)	3 (16.7)	4 (22.2)	0 (0.0)
**Grade 8**	10	0 (0.0)	1 (10.0)	9 (90.0)	0 (0.0)	1 (10.0)	0 (0.0)
**School term**							
**Term 1**	134	3 (2.2)	6 (4.5)	103 (76.8)	18 (13.4)	4 (3.0)	3 (2.1)
**Term 2**	102	0 (0.0)	3 (2.9)	83 (81.4)	16 (15.7)	3 (2.9)	1 (0.9)
**Term 3**	148	8 (5.4)	6 (4.0)	102 (68.9)	13 (8.8)	28 (18.9)	1 (0.7)
**Symptoms**							
**Cough**	287	8 (2.8)	10 (3.5)	215 (74.9)	40 (13.9)	30 (10.4)	3 (1.0)
**Runny nose**	346	9 (2.6)	14 (4.1)	260 (75.1)	41 (11.9)	30 (8.7)	5 (1.4)
**Sore throat**	75	1 (1.3)	3 (4.0)	58 (77.3)	6 (8.0)	7 (9.3)	1 (1.3)
**Class size**							
**<45 students**	161	3 (1.9)	5 (3.1)	122 (75.8)	24 (14.9)	14 (8.7)	2 (1.20)
**>45 students**	223	8 (3.6)	10 (4.5)	166 (74.4)	23 (10.2)	21 (9.4)	3 (1.2)
**BMI**							
**Underweight**	81	1 (1.2)	5 (6.2)	61 75.3)	12 (14.8)	5 (6.2)	2 (2.3)
**Normal**	297	9 (3.3)	9 (3.0)	222 (74.8)	34 (11.4)	30 (10.1)	3 (0.9)
**Overweight**	6	1 (16.7)	1 (16.7)	5 (83.3)	1 (16.7)	0 (0.0)	0 (0.0)
*BMI:- Body Mass Index							

Comparison of virus proportions in the different grades identified rhinovirus and coronavirus as the most prevalent viruses detected in samples from each of the 12 grades in the school. Influenza virus was the least commonly detected virus found circulating in only 33.3% (4/12) of the grades in the school and in 1.5 % of the students who were sampled. The distribution of respiratory virus detections in the different grades is shown in Table 4.

**Table 4.** Detections in grades, students and samples, by virus target, in a rural school in coastal Kenya, year 2017–18.

Description	Grades, N=12 n (%)	Students (N=325) n (%)	Samples, N=1726 n (%)
**Any virus**	12 (100)	176 (54.2)	384 (22.2)
**Rhinovirus**	12 (100)	147 (45.2)	288 (75.0)
**Adenovirus**	9 (75)	13 (4.0)	15 (0.9)
**Human coronavirus**	12 (100)	31 (9.5)	35 (2.0)
** OC43**	7 (58)	12 (3.7)	12 (0.7)
** NL63**	9 (75)	15 (4.6)	17 (1.0)
** 229E**	5 (42)	6 (1.8)	6 (0.4)
**Parainfluenza viruses**	11 (92)	41 (12.6)	47 (2.7)
** PIV2**	7 (58)	13 (4.0)	14 (0.8)
** PIV3**	10 (83)	23 (7.1)	24 (1.4)
** PIV4**	7 (58)	10 (3.1)	10 (0.6)
**Respiratory syncytial virus** **(RSV)**	7 (58)	11 (3.4)	11 (0.6)
** Group A**	5 (42)	8 (2.50	8 (0.5)
** Group B**	3 (25)	3 (0.9)	3 (0.2)
**Influenza virus**	4 (33)	5 (1.5)	5 (0.3)
** Type A**	4 (33)	5 (1.5)	5 (0.3)
** Type B**	1 (8)	1 (0.3)	1 (0.1)
** Type C**	1 (8)	1 (0.3)	1 (0.1)

The crude proportion of virus positive samples from smaller sized classes, less than the Kenyan recommended average class size of 45 students, was higher compared to the larger classes of >45 students, 27.3% vs 19.6% respectively *χ ^2^* = 13.8, P <0.001.

### Respiratory virus detections by age

**Figure 2.**
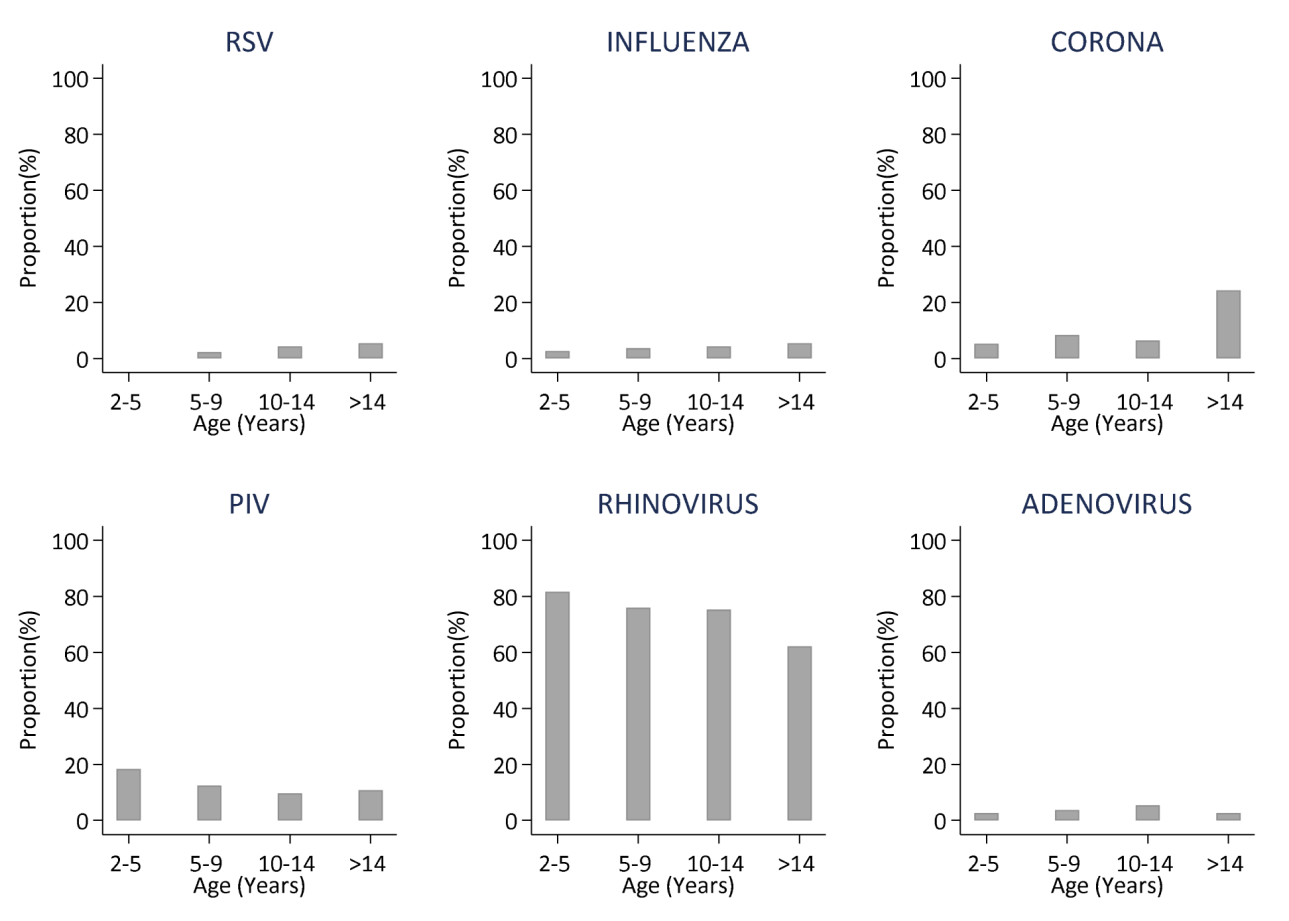
Distributions of the six different virus groups detected in samples collected from school children over a one school year stratified by age. Proportions (%) are the number positive out of the number of samples collected, by age group., (RSV- respiratory Syncytial Virus; FLU- Influenza virus A, B, C; PIV -Parainfluenza virus 1–4; Corona- Human coronavirus NL63,E229,OC43; Rhinovirus-Human rhinovirus).

The distribution of respiratory viruses by age is shown in Figure 2. Influenza virus, coronavirus, parainfluenza virus and rhinovirus were detected across all age groups. There were no RSV detections in samples from students aged below 5 years and those above 14 years, respectively. The prevalence of coronavirus was highest in samples obtained from students above 14 years. Though not statistically significant, the proportions of rhinovirus and parainfluenza virus was higher in samples from students below 5 years compared to the older age groups ( Table 3).

**Figure 3.**
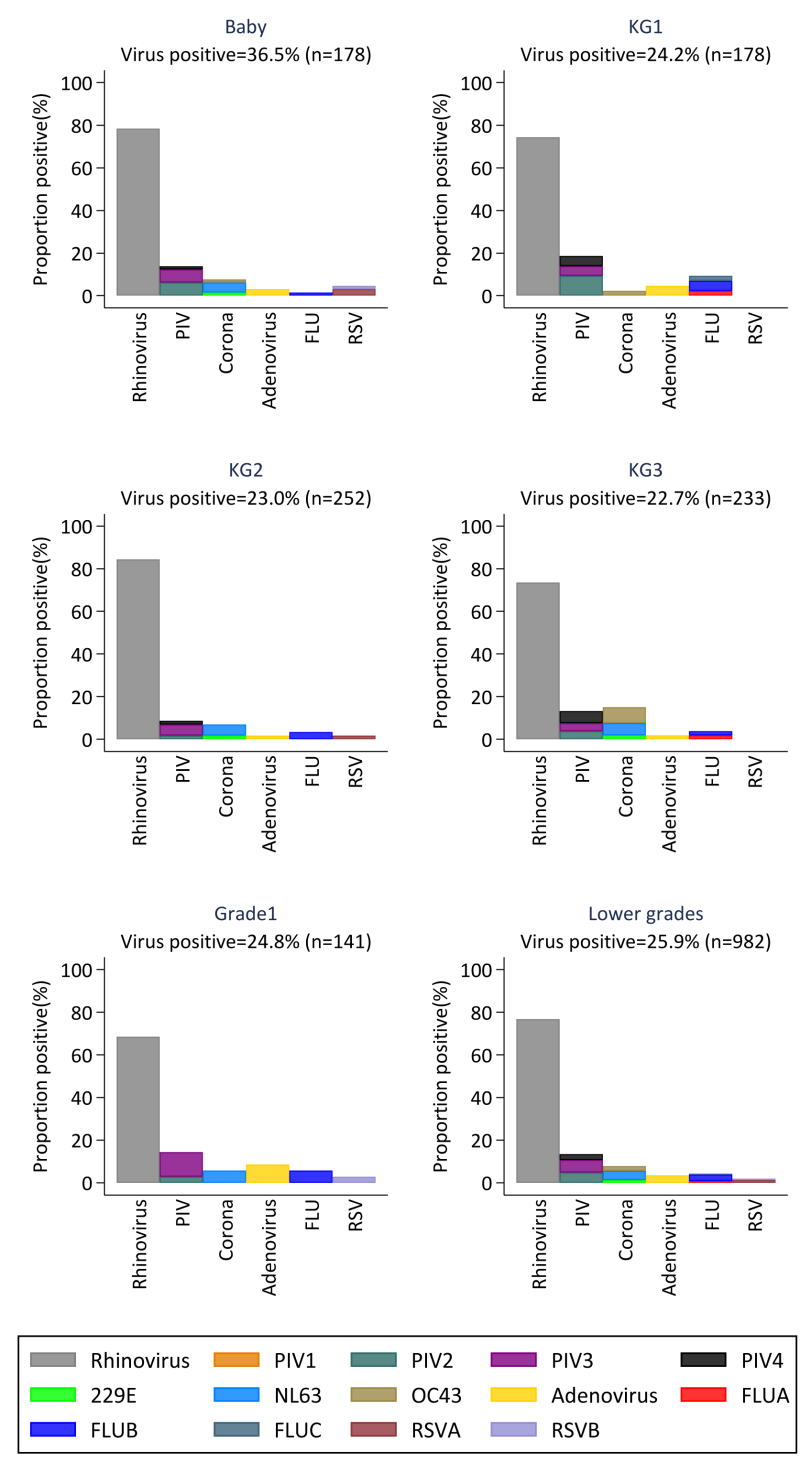
Proportions of virus positive samples in each of the grades in lower primary. Each panel shows the distribution of the six different virus groups per grade. (RSV- respiratory Syncytial Virus; FLU- Influenza virus A, B, C; PIV -Parainfluenza virus 1–4; Corona- Human coronavirus NL63, E229, OC43; Rhinovirus-Human rhinovirus).

**Figure 4.**
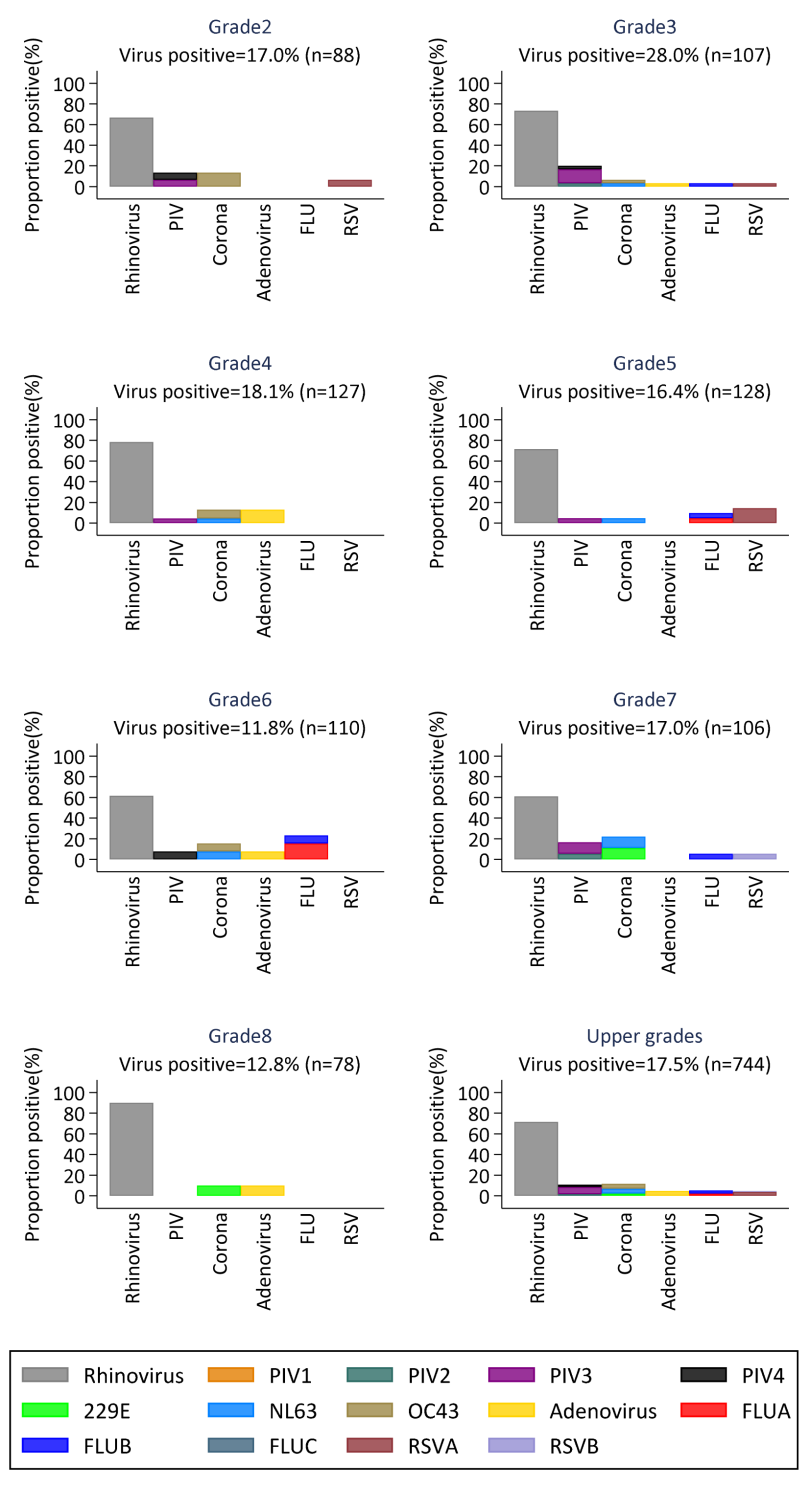
Proportions of virus positive samples in each of the grades in upper primary. Each panel shows the distribution of the six different virus groups per grade. (RSV- respiratory Syncytial Virus; FLU- Influenza virus A, B, C; PIV -Parainfluenza virus 1–4; Corona- Human coronavirus NL63, E229, OC43; Rhinovirus-Human rhinovirus).

The proportion of viruses circulating in the lower and upper primary grades during the study period is shown in Figure 3 and Figure 4, respectively. Daycare children (labelled “Baby”) had the highest proportion of virus positive samples compared to the other lower primary children. Except for RSV which was only detected in three of the lower primary grades, all other virus groups were detected at least once in all lower primary grades. In the upper grades, only rhinovirus and coronavirus were detected in all grades, and grade 3 was the only grade in which all six virus groups were detected at least once during the study period.

### Coinfections

There was a median of 1 (range, 1–3) virus detected per sample. Twenty-nine (7.6%) of the 384 positive samples had more than one virus detected. Amongst samples with more than one virus detected, 27 had 2 viruses, and 2 had 3 viruses co-detected. Eighteen (62.1%) of the samples with co-detection of viruses were from children in the lower primary grades. Rhinovirus was detected in 19 samples with co-infections, parainfluenza viruses in 15 samples, coronaviruses in 7 samples, adenovirus and influenza viruses in 5 samples each and RSV in 3 samples. Rhinovirus co-infected at least once with all other virus target groups.

### Seasonality

**Figure 5.**
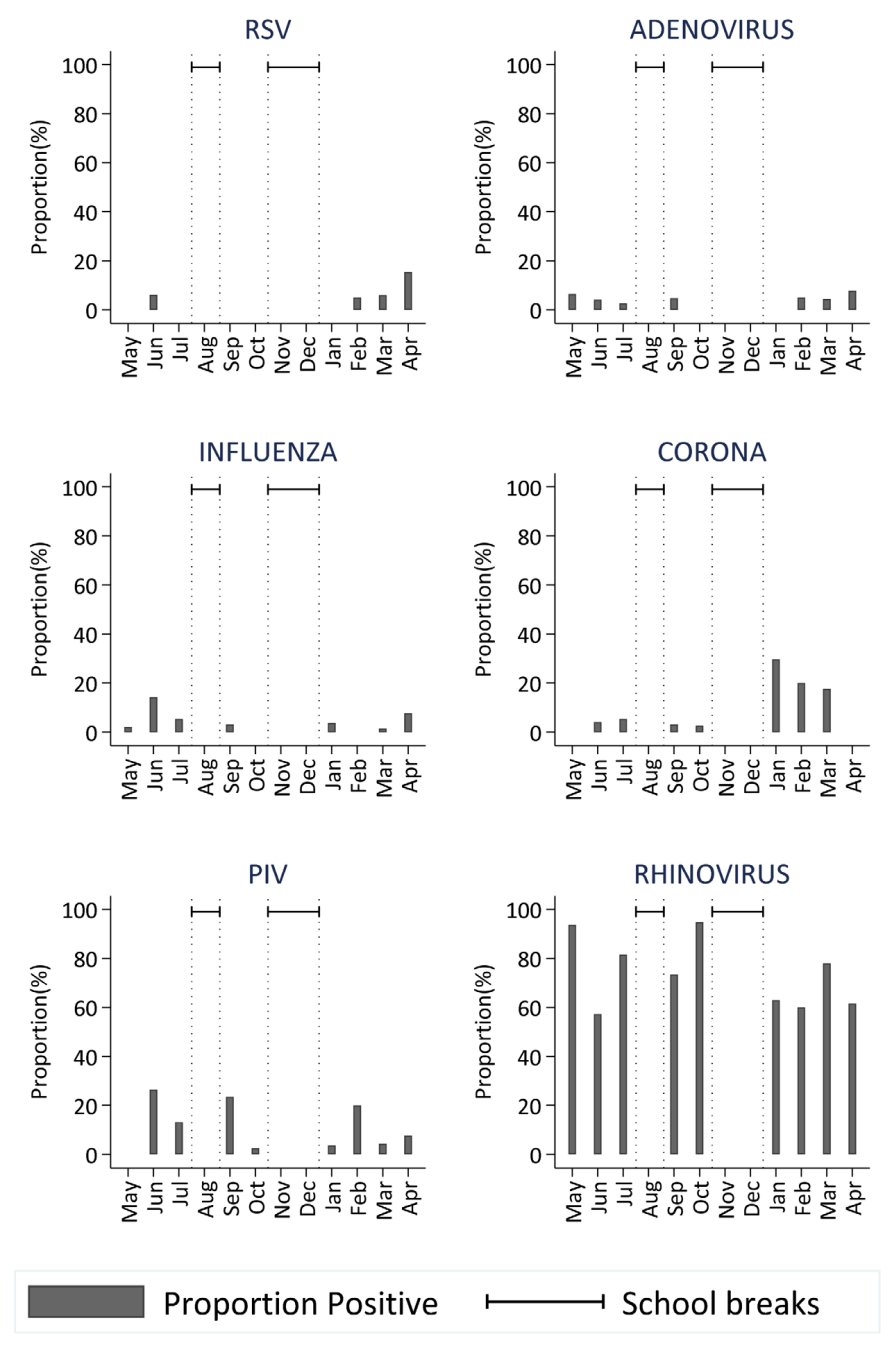
Temporal distribution of the proportion of virus-positive nasopharyngeal swab samples over the period May 2017 to April 2018 (with school breaks indicated) for the six virus groups obtained during the surveillance of ARI in the primary school. (RSV- respiratory Syncytial Virus; FLU- Influenza virus A, B, C; PIV -Parainfluenza virus 1-4; Corona- Human coronavirus NL63, E229, OC43; Rhinovirus-Human rhinovirus)

There was at least one respiratory virus in circulation in the school during all the months of the study. Rhinovirus was detected during all the months when the school was in session with no distinct peaks. Coronavirus and parainfluenza virus were detected during 90% of the months with peaks in the months of January and June, respectively. RSV and influenza viruses were the least commonly detected viruses. RSV was detected during the first term of the school year, a period coinciding with the RSV epidemics in the community ( Figure 5).

### Comparison of virus detections in the school, community and hospital

**Figure 6.**
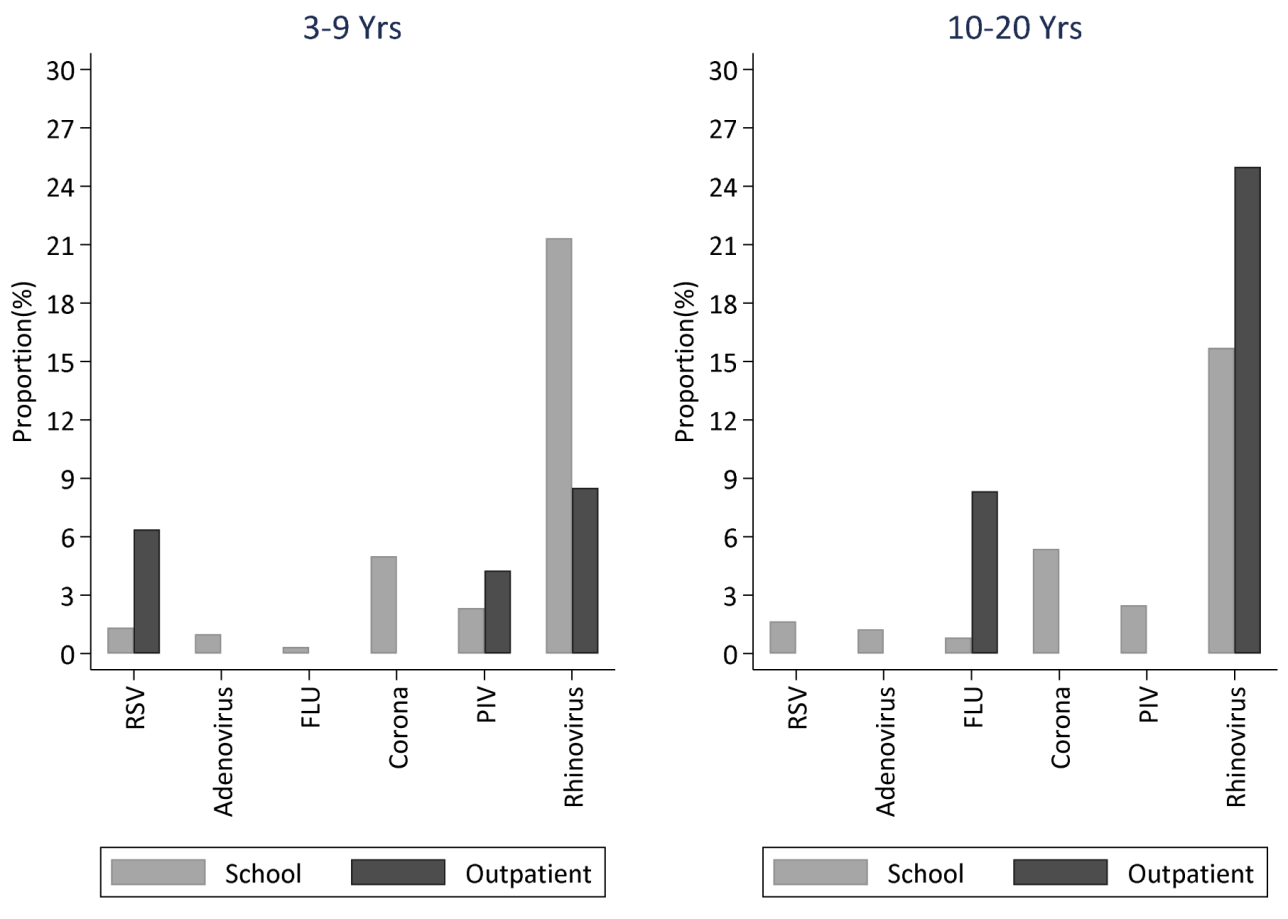
Comparison of virus surveillance between the school setting and the local outpatient facility for children aged between 3–9 years and 10–20 years. The left and right panels shows a comparison of the proportion of positive nasopharyngeal samples positive for each of the six virus groups in the school and outpatient facility among children aged 3–9 years and 10–20 years, respectively . (RSV- respiratory Syncytial Virus; FLU- Influenza virus A, B, C; PIV -Parainfluenza virus 1-4; Corona- Human coronavirus NL63, E229, OC43; Rhinovirus-Human rhinovirus).

A comparison of viruses detected in the school and the nearest outpatient clinic surveillance in participants aged 3–20 years during the first school term in 2018 is shown in Figure 6. Like the school setting, rhinovirus was the most commonly detected virus in samples collected from presenting ARI patients in the health Centre.

**Figure 7.**
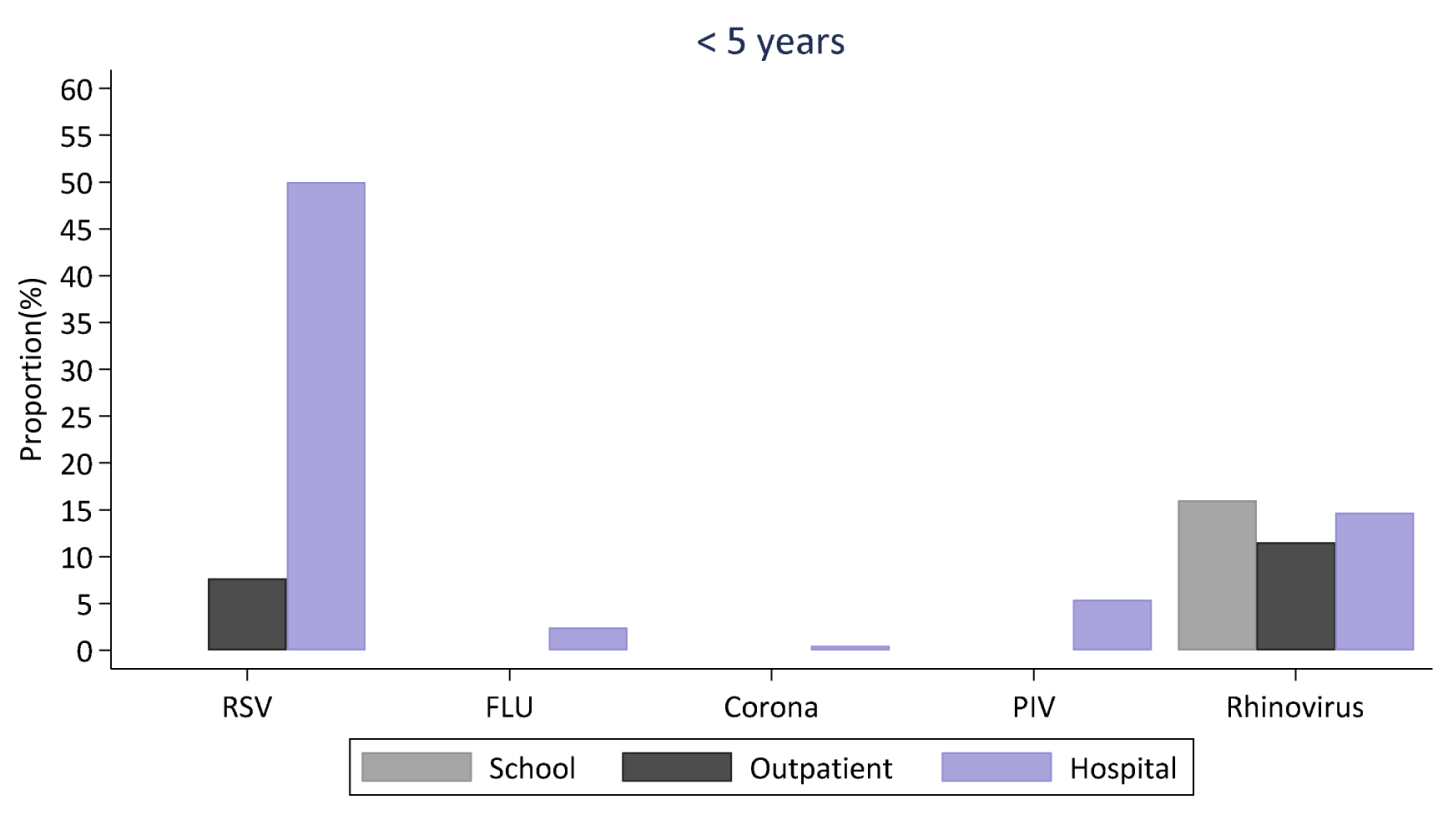
Comparison of virus surveillance between the school setting, the local outpatient facility and inpatient hospital for children below five years.

Overall, more virus groups were detected in samples from the school compared to samples from the local health facility. However, the proportion of RSV and parainfluenza viruses was higher in the outpatient compared to the school setting in children aged 3–9 years. Only three virus types (rhinovirus and influenza virus A and B) were detected in individuals aged 10–20 years in the outpatient compared to six virus types detected in students aged 10–20 years in school. A comparison of the distribution of viruses in the school, outpatient and inpatient hospital setting within the same location among children below 5 years during the same first school term of 2018 is shown in Figure 7. Rhinovirus was detected in all three settings and it was the only virus detected among children below 5 years in the school. RSV was the most commonly detected virus among the hospital cases.

## Discussion

School-going children have previously been identified as a key group in the spread of respiratory infections in the community, including into the household ^19, 33, 34^. With regard to respiratory virus transmission, it is asserted that school age children play a major role in the early stages of an epidemic and contribute extensively to the spreading of the virus in the population ^33^. Little has been done to critically investigate the mechanisms of transmission of respiratory viruses in schools and to quantify the extent to which they contribute to the transmission of respiratory viruses in the community. The few studies conducted in this area have focused on the prevalence of ARIs in school children, normally based on reported symptoms and data collected at a single point in time ^35, 36^. To gain more insight into the dynamics of transmission we conducted a one-year surveillance of respiratory viruses across all grades in a school setting, collecting nasal samples weekly from symptomatic students and screening them for respiratory viruses. To the best of our knowledge, this is the first study of its kind in sub-Saharan Africa.

Following an intense period of community engagement and sensitization, about 62% of the school population was recruited into the cohort and followed up for a period of one school year. Data from some of the studies conducted in the KHDSS show that the response rate in our study was similar to what has been observed in previous research studies in the area, attributed partly to the intense nature of the study and research fatigue ^37^.

Results from the surveillance showed presence of respiratory viruses in circulation in the school all year round with 86% of the students in the cohort experiencing mild respiratory symptoms at least once during the study period. Approximately 22% of the samples had at least one respiratory virus detected. A similar study among pre-school and primary school age children (3–14 years) in Israel showed higher proportions of virus positive samples (49–57%) ^38^. This could however be attributed to the fact that the participants in that study were children attending pediatric clinics presenting with upper respiratory tract infection symptoms including fever, a factor which might have preselected more severe and likely more virus positive cases compared to the mild cases identified in our study. 

In the present study, the proportion of virus positive samples was highest among the students below 5 years despite having low numbers of students of this age group in the cohort. This is in line with global statistics which indicate the burden of ARIs to be highest in children below 5 years old. Younger students aged 5–9 years in lower grades also had a significantly higher proportion of virus positive samples compared to the older students (≥10yrs), a finding consistent with household studies that showed lower prevalence and lower risk of respiratory virus infection in older children ^19, 34, 39^.

The proportion of virus positive samples was higher among male students compared to female students. A study on prevalence of infections among 6–16 year old children in India ^35^ showed similar results where boys had more frequent upper respiratory tract infection episodes compared to girls. The results are however not conclusive since other prevalence studies in school-going children found no association between gender and respiratory infection episodes ^36, 40^.

Like other studies investigating ARIs in children, rhinovirus was found to be the most frequently detected virus. This was in accord with community and outpatient clinic studies where rhinovirus was the predominant virus detected among preschool and primary school-age children especially immediately after opening of schools ^24, 38, 41, 42^. Rhinovirus showed no seasonality and was detected during all the months of the study without any distinct peaks. Rhinovirus is known to cause upper respiratory disease which is mild in nature but has also been associated with severe lower respiratory infections which can lead to hospitalizations ^38, 43^.

Coronavirus was among the frequently detected viruses in our study detected in samples from all the 12 grades in the school. Occurrence of the virus was seasonal, with a peak in the month of January and most detections during the first term of the year, a finding not consistent with a community-based study in the same region ^22^. A three-year surveillance study in the US identified NL63 as the most prevalent species, as was observed in our study. NL63 has also previously been associated with lower respiratory infections ^42, 44^. Other studies have found OC43 to be more prevalent ^45^.

Influenza viruses were uncommon in all age groups. Similarly, within this coastal Kenya setting influenza is not a frequent cause of hospitalized pediatric pneumonia ^1, 46^ (also Figure 7). Furthermore, in the ARI presentations to the local health facility ( Figure 6), influenza was absent from samples from the younger age group (3–9 years), although 9% of samples were positive in the 10–20 year age group.

It was surprising not to detect any RSV among children aged less than 5 years, and only a few detections among older age groups (>5 years) over the entire study period. This observation contravenes our perception that schools are a major hub in the transmission of RSV in the community ^19^. However, this observation needs to be viewed with caution as the likely explanation might be related to our sampling strategy. First, given RSV is known to cause more severe ARI in these age groups ^22, 24^, the severely ill would have been missed through our school-based sampling strategy, and would therefore not have been captured during the weekly sampling strategy. Furthermore, our sampling strategy was constrained by adhering to a fixed sample size per grade, which may have contributed to lower detections of RSV. 

Rates of respiratory viral coinfections vary appreciably in different studies with different subjects. It was previously presumed that coinfection was a predictor of respiratory disease severity in children. Presence of coinfections in samples from mild disease in our study is in agreement with the conclusion that coinfections should not necessarily be considered a proxy of clinical severity ^47– 49^.

The recommended class size in Kenyan primary schools is 45 students per class ^50^. Results from our study show that children in larger classes had proportionately fewer respiratory virus infections compared to those in smaller classes ( Table 2), suggesting that large class sizes are protective. In our study there was a trend between class size and age where larger classes had older students. This relationship between class size and respiratory virus infections may arise from factors such as increased awareness of hygiene, level of immunity and heterogeneity of mixing within classes which increase with age. Upon further analysis we found that the association between class size and infection was confounded by students age.

We did not have enough data to fully characterize virus seasonality. However, some viruses were detected during specific times of the year. RSV was detected during the first quarter of the year. This is consistent with other surveillance findings from this community ^22^.

Rhinovirus is seen to cause both mild and moderate illness as it was the predominant virus detected in both the school and outpatient settings across all age groups. RSV was notably higher in the inpatient hospital compared to school settings. 

The study had a number of limitations. First, we did not collect nasal samples from all symptomatic students. We randomly selected a maximum of 8 symptomatic students in the lower primary and a maximum of 4 symptomatic students in the upper primary to give nasal samples every week. Further, samples were usually collected on a single day of the week. This would result in an underestimation of the full burden of respiratory infections. It also means that the seasonal patterns of one virus is not independent of another, since changes in prevalence of one virus would alter the likelihood of identifying other viruses. Second, we had many students (149/325) with ARI symptoms sampled but turned out negative for all respiratory pathogens screened. The lack of adequate data on family history of allergic rhinitis could have resulted in a larger number of students identified with “ARI symptoms” which were not caused by any viral pathogen. Subsequently this might have caused an underestimate of the viral detections by diluting the pool of truly ARI symptomatic students and reducing chances of selecting students with virus infection for sample collection. Third, there was a high probability that some students with moderate to severe symptoms could have stayed at home and been missed during sampling of symptomatic students. This could have biased our estimation of viral infections circulating in the school to those associated with mild symptoms. Fourth, we only collected nasopharyngeal swabs, and it is known that the addition of an oropharyngeal swab can increase the detections of some viruses, e.g. influenza, parainfluenza and adenovirus ^51^. Finally, our symptom inclusion criteria did not include measured fever as for ILI (influenza like illness) which also may have reduced the frequency of some viruses. Nonetheless, our study has important strengths, including that symptoms data were collected, longitudinal testing was conducted, and a sensitive multiplex real-time assay was used to detect the targeted viruses.

In conclusion, our findings from the one-year surveillance confirm that multiple respiratory viruses circulate in school populations and primary school children suffer numerous episodes of mild viral respiratory infections all year round. Rhinovirus was observed to be the dominant virus in ARI presentations in the school setting as in the outpatient setting. Our study provides an important first layer towards understanding the transmission dynamics of respiratory infections in school aged children and the role of the school environment in the transmission of viral respiratory infections in communities. To determine the key drivers of transmission, further studies that link the data from samples to contact patterns between the school children and further to the households are required to answer the question of “Who Infects Whom?”. In addition, our study confirms that school-based surveillance of viral respiratory infections is feasible. School based surveillance can allow tracking of emerging respiratory infections and form a basis of initiating school-level interventions. Future studies in this population should also investigate modifiable risk factors that could be targets of interventions towards prevention of viral respiratory infections.

## Data availability

### Underlying data

Harvard Dataverse: Replication Data for: Surveillance of respiratory viruses among children attending a primary school in rural coastal Kenya. https://doi.org/10.7910/DVN/AAA4JN ^27^

This project contains the following underlying data:

- Datasets_csv_files.zip (contains the main datasets used in the analysis including data on PCR results, anthropometric measures, data on samples collected from the outpatient facility and data on samples collected from the inpatient records, .csv format)
- Datasets_stata_files.zip (contains the main datasets used in the analysis including data on PCR results, anthropometric measures, data on samples collected from the outpatient facility and data on samples collected from the inpatient records, .dta format)
- IAdema_Spred_schools_codebook.pdf (contains a file describing all the study variables)
- IAdema_Spred_Schools_ReadMe.txt (contains the Main project summary)
- Scripts.zip (contains the scripts and STATA do files used in analysis of the data)

### Extended data

Harvard Dataverse: Replication Data for: Surveillance of respiratory viruses among children attending a primary school in rural coastal Kenya. https://doi.org/10.7910/DVN/AAA4JN ^27^

This project contains the following extended data:

- Flu Register (contains the study questionnaire)
- SPReD_bmi_calculator_metric (the guide for calculating the BMI for age scores)

Data are available under the terms of the Creative Commons Attribution 4.0 International license (CC-BY 4.0).
