# Supplementary material for: Surveillance of respiratory viruses among children attending a primary school in rural coastal Kenya
Source: Wellcome Open Res. 2020 Sep 24;5:63. Originally published 2020 Apr 6. [Version 2] doi: 10.12688/wellcomeopenres.15703.2 (PMC7569485; doi:10.12688/wellcomeopenres.15703.2)
Supplement: Peer review history

# Peer review: Reviewer response for version 2

**DOI:** 10.21956/wellcomeopenres.17941.r40622

**Published:** 2020-10-13

**Authors:** David P. Moore

**Affiliations:** 1 Medical Research Council, Respiratory and Meningeal Pathogens Research Unit, University of the Witwatersrand, Johannesburg, South Africa; 2 Department of Paediatrics and Child Health, Chris Hani Baragwanath Academic Hospital, University of the Witwatersrand, Johannesburg, South Africa

Thank you for the opportunity of reviewing the revised manuscript, which has been strengthened through adoption of suggestions made in the initial round of peer review.

 There are two minor edits that could be adopted:

1. Under "Sampling" (1st sentence), reword as "... daily "in seven-day symptom diaries which..."
2. Under "Sampling" (4th sentence), reword as "... checking their symptom diaries..."

Is the work clearly and accurately presented and does it cite the current literature?

Yes

If applicable, is the statistical analysis and its interpretation appropriate?

Yes

Are all the source data underlying the results available to ensure full reproducibility?

Yes

Is the study design appropriate and is the work technically sound?

Yes

Are the conclusions drawn adequately supported by the results?

Yes

Are sufficient details of methods and analysis provided to allow replication by others?

Yes

Reviewer Expertise:

Paediatric infectious diseases, particularly pneumonia.

I confirm that I have read this submission and believe that I have an appropriate level of expertise to confirm that it is of an acceptable scientific standard.

# Peer review: Reviewer response for version 2

**DOI:** 10.21956/wellcomeopenres.17941.r40621

**Published:** 2020-09-30

**Authors:** Ting Shi

**Affiliations:** 1 Centre for Global Health, Usher Institute, University of Edinburgh, Edinburgh, UK

I have no further comments. The revised version is more structured and clearer, especially in the methods part.

Is the work clearly and accurately presented and does it cite the current literature?

Yes

If applicable, is the statistical analysis and its interpretation appropriate?

Yes

Are all the source data underlying the results available to ensure full reproducibility?

Yes

Is the study design appropriate and is the work technically sound?

Yes

Are the conclusions drawn adequately supported by the results?

Yes

Are sufficient details of methods and analysis provided to allow replication by others?

Yes

Reviewer Expertise:

epidemiology, respiratory viral infection

I confirm that I have read this submission and believe that I have an appropriate level of expertise to confirm that it is of an acceptable scientific standard.

# Peer review: Reviewer response for version 1

**DOI:** 10.21956/wellcomeopenres.17212.r38362

**Published:** 2020-06-05

**Authors:** Ting Shi

**Affiliations:** 1 Centre for Global Health, Usher Institute, University of Edinburgh, Edinburgh, UK

This article presents in detail on the epidemiology of circulating respiratory viruses among students from a primary school in rural coastal Kenya. The findings are very comprehensive. I have a few questions.

1. What about asymptomatic students with ARI? Did you test them?
2. Can you provide the details on the underlying medical conditions among the included students?
3. 405 students had ARI symptoms but samples were taken from 325 of them. Can you explain why? You also mentioned in earlier paragraph that 101 samples were excluded from analysis. Can you provide the reason for this? What influence this might have?
4. What happened to these students with ARI? Did any of them seek hospital care? Were any of them admitted to hospitals? I am also worried that students with severe symptoms perhaps wouldn't come to school and you only had students with mild symptoms.
5. Can you describe a bit more on co-infection? Did them present differently to single infection? Did them tend to have different severity?
6. Do you expect to see different patterns of the circulating viruses during school breaks?
7. Can you provide some discussions on the bias and confounding from this project?

Is the work clearly and accurately presented and does it cite the current literature?

Yes

If applicable, is the statistical analysis and its interpretation appropriate?

Yes

Are all the source data underlying the results available to ensure full reproducibility?

Yes

Is the study design appropriate and is the work technically sound?

Yes

Are the conclusions drawn adequately supported by the results?

Yes

Are sufficient details of methods and analysis provided to allow replication by others?

Yes

Reviewer Expertise:

epidemiology, respiratory viral infection

I confirm that I have read this submission and believe that I have an appropriate level of expertise to confirm that it is of an acceptable scientific standard, however I have significant reservations, as outlined above.

# Response

**Published:** 2020-09-15

**Authors:** Irene Adema Wangwa

**Affiliations:** KEMRI Wellcome Trust Research programme, Kenya

**Responses to comments from reviewer 2**

This article presents in detail on the epidemiology of circulating respiratory viruses among students from a primary school in rural coastal Kenya. The findings are very comprehensive. I have a few questions.

**1. What about asymptomatic students with ARI? Did you test them?** *In this study, we focused on students who had symptoms of ARI specifically cough, nasal discharge and sore throat. Samples were not collected from asymptomatic students and thus they were not tested.*

**2. Can you provide the details on the underlying medical conditions among the included students?** *The study focused on the surveillance of respiratory viruses circulating among the student population. All the participants who gave a sample had mild symptoms. We did not get details of the underlying medical conditions among the students who gave a sample as they did not show signs of severe disease.*

**3. 405 students had ARI symptoms but samples were taken from 325 of them. Can you explain why? You also mentioned in earlier paragraph that 101 samples were excluded from analysis. Can you provide the reason for this? What influence this might have?** *Study procedures required that students to keep a daily symptoms diary in which they would self-report any ARI symptoms they experienced on a daily basis. Results from analysis of the symptom diaries are presented in a separate manuscript in preparation. Every week,* *Only children considered to be appropriately aged for their school grade are included in this analysis. Those >10 years in lower primary were considered too old for their grade, while those aged <10 years and in upper primary were considered too young for their grade. Results from NPS samples obtained from those children (101 samples), as well as the results of samples obtained from school teachers, will be presented in separate manuscripts.*

**4. What happened to these students with ARI? Did any of them seek hospital care? Were any of them admitted to hospitals? I am also worried that students with severe symptoms perhaps wouldn't come to school and you only had students with mild symptoms** *All students in the study who gave a sample had mild symptoms. For majority of those who were sampled, symptoms resolved without progressing to severe illness. Any student whose symptoms progressed from mild to severe visited the local health centre for treatment and did not attend school until they had recovered. We do not have details of whether any of the students were admitted to hospital due to ARI. Students who developed severe symptoms while at school during the study period were taken to the local health centre for treatment by their teachers. Nasal samples were not collected from the students on that day. Students who visited the local health centre could have been sampled as part of the community surveillance which was conducted at the hospital during the same period*.

**5. Can you describe a bit more on co-infection? Did them present differently to single infection? Did them tend to have different severity?** *There was no difference in presentation of symptoms between single infections and co-infections. All students whose samples were found to have co-infections had mild symptoms. Occurrence of multiple respiratory viruses infection did not change severity of symptoms among the students*.

**6. Do you expect to see different patterns of the circulating viruses during school breaks?** *In this study we did not follow the students for surveillance of ARI during the school breaks. For this reason, we are unable to determine if the pattern of circulating viruses is different when the school was in session and during the school breaks. Community surveillance during the school breaks might be able to shed some insights into this.*

**7. Can you provide some discussions on the bias and confounding from this project?** *There were a few biases identified in the study. These are mentioned and discussed in the manuscript as study limitations. The study could have suffered selection bias due to the sampling regime used. Although simple randomization was used to select those who gave a sample, there is a likelihood of underestimation of the quantity of circulating viruses because we used a convenient sample size.*

*We did not collect information on most of the other known risk factors to enable us to analyze for all confounders. We however identified age as a confounder of the association between class size and ARI infection. The study was mainly observational aiming at describing the nature of respiratory viruses circulating in a rural school setting. More research is needed to determine the risk factors for ARI in school children.*

# Peer review: Reviewer response for version 1

**DOI:** 10.21956/wellcomeopenres.17212.r38605

**Published:** 2020-05-05

**Authors:** David P. Moore

**Affiliations:** 1 Medical Research Council, Respiratory and Meningeal Pathogens Research Unit, University of the Witwatersrand, Johannesburg, South Africa; 2 Department of Paediatrics and Child Health, Chris Hani Baragwanath Academic Hospital, University of the Witwatersrand, Johannesburg, South Africa

Thank you for the opportunity of reviewing this well-presented article from an established public health research group in Kilifi. The research centres on serial testing of children symptomatic with acute respiratory infection at a primary school, and documents the prevalence of circulating respiratory viruses in a school year. Unsurprisingly, human rhinovirus was the most frequently detected respiratory virus during the course of the study.

 Major comment:

- The authors mention that a limitation of their study was that they were unable to sample all symptomatic children in the study. This is important, but needs to be elaborated on. What proportion of all symptomatic children in each grade were sampled? Were absenteeism lists maintained throughout the study to give an indication of the proportion of children that missed school days due to illness? Such information would assist in enabling a fuller understanding of the representativeness of the results achieved in this study.

Minor comments:

1. Suggest exclude the first two sentences of the Results section, and reposition them in the Methodology section, at the end of the second paragraph under the subheading “Sampling”.
2. Check the math in the percentage of scholars included in the study: 469/781 = 60.1% = 60%.
3. Suggest exclude the sentences starting with “Students aged >10 years and in lower primary…” through to the end of the first paragraph of the Results section. Rather include these as exclusion criteria for presentation in this analysis, in the Methods section. I think that the best place to reposition a modified presentation of this material would be at the end of the second paragraph under the subheading “NPS sample and data collection”. Consider rewording as: “Only children considered to be appropriately aged for their school grade are included in this analysis. Those >10 years in lower primary were considered too old for their grade, while those aged <10 years and in upper primary were considered too young for their grade. Results from NPS samples obtained from those children, as well as the results of samples obtained from school teachers, will be presented in separate manuscripts.”
4. Check the math in the percentage of included children that were female: 253/469 = 53.9% = 54%.
5. Check the math in the percentage of included children that were in Grade 1: 72/469 = 15.4%.
6. This analysis expressly stated that children too old or young for their schooling tier were excluded: the median ages of children included in the analysis must be recalculated, and the range presented as maximum 9.9 years in the lower tier, range presented as minimum 10 years in upper tier.
7. Suggest exclude the words “in detail” in the last sentence of the second paragraph of the Results section.
8. The under sampling of children in kindergarten, and in those <5 years of age in this study runs counter to the anticipated finding of the highest rate of ARI in this group of children.
9. Check the math in the percentage of samples that tested positive for at least 1 virus: 384/1726 = 22.2%.
10. In the second paragraph under the heading “Virus detections from NPS samples”, please correct the percentage of swabs positive in the upper primary students to 17.5%; also, please add in a percentage sign for “25.9%” of swabs positive in the lower primary.
11. In the second paragraph under the heading “Virus detections from NPS samples”, please reword as “… male students had a higher proportion of virus positive swabs compared to…”
12. In the first sentence of the third paragraph under the heading “Virus detections from NPS samples”, please correct to “… contributing to 7.4% of collected samples.”
13. In the second sentence of the third paragraph under the heading “Virus detections from NPS samples”, please correct to “… (29.9%, 38/127)…”
14. In the first sentence of the fourth paragraph under the heading “Virus detections from NPS samples”, suggest rephrase as “… sample was positive from HMPV or parainfluenza virus type 1.”
15. It is unclear how the authors derived the proportion of students sampled that were positive for rhinovirus in the fourth paragraph under the heading “Virus detections from NPS samples”. The current attribution is 54.2% of sampled scholars were positive for rhinovirus; however this seems to conflict with numbers presented in Table 4, in which 147 (45.2%) of 325 students tested positive for rhinovirus. Please correct as appropriate.
16. In the last sentence of the fourth paragraph under the heading “Virus detections from NPS samples”, the authors should present the significance of the association between RSV and school term using Fisher’s exact test, rather than the Chi square test.
17. In the first paragraph under the heading “Respiratory virus detection by age”, the authors contend that rhinovirus and parainfluenza virus were “notably” more prevalent in children <5 years of age, but this is not borne out by statistical analysis of the available data. Suggest rephrase to indicate that these findings were not statistically significant.
18. In Figure 1, suggest reword the subcaption as “Virus positive (384/1726, 22.2%)”.
19. Figure 2 adds no extra information beyond Table 3: suggest exclude.
20. In the first paragraph under the heading “Respiratory virus detection by age”, the authors state that no adenovirus was detected in children >14 years of age, but this assertion conflicts with data presented in Table 3 and in Figure 2 where one child ≥15 years had an adenoviral detection. Please correct.
21. In the second paragraph under the heading “Respiratory virus detection by age”, the authors contend that daycare attendees had the highest proportion of positive samples compared to other lower primary attendees. This is not immediately evident from Figure 3 (in which it is unclear which of the bar charts reflect daycare attendees specifically). Also, no attempt is made at conducting a formal statistical analysis to compare the prevalence of virus detection in each group. Suggest include such a comparison, and clarify for the reader.
22. The wording of the last sentence of the second paragraph under the heading “Respiratory virus detection by age” is somewhat confusing, as it is not immediately clear that the shift in focus from lower to upper grades has occurred until midway through the sentence. Suggest reword as: “In the upper grades, only rhinovirus and coronavirus were detected in all grades, and grade 3 was the only grade in which all six virus groups were detected at least once during the study period.”
23. Suggest reword the paragraph under the heading “Coinfections” as: “There was a median of 1 (range, 1-3) virus detected per sample. Twenty-nine (7.6%) of the 384 positive samples had more than one virus detected. Amongst samples with more than one virus detected, 27 had 2 viruses, and 2 had 3 viruses co-detected. Eighteen (62.1%) of the samples with co-detection of viruses were from children in the lower primary grades. Rhinovirus was detected in 15 samples, …”
24. In Table 3, close off the parentheses in the last column in the row labelled as “<45 students”.
25. The short paragraph on the finding that more viral infections were found in small-sized classes rather than larger classes seems out of place at the end of the section under the heading “Seasonality”. Suggest move this to the end of the section headed as “Virus detections from NPS samples”.
26. It is useful to have the comparisons between the school environment and outpatient setting, which highlights important differences in the prevalence of viral detection between the settings. I would encourage the authors to go a step further and do a formal statistical comparison of detection rates, and to reflect the statistical significance of the findings in Figures 6 and 7.
27. In the fourth sentence of the first paragraph of the Discussion, suggest reword as: “… normally based on reported symptoms and data collected at a single point in time ^35, 36^.”
28. In the second paragraph of the Discussion, suggest reword as: “… that the response rate…”
29. In the paragraph on coronavirus detection in the Discussion section, suggest add in a comma to clarify the meaning of the sentence, like this: “… most prevalence species, as was…”
30. Note that coronavirus NL63 is a species, not a strain, of virus.
31. In the paragraph describing influenza detection in the Discussion, suggest reword as: “… although 9% of samples were positive in the 10-20 year age group.”
32. In the paragraph on RSV detection in the Discussion, suggest reword as: “… would have been missed through our school-based sampling strategy, and would therefore not have been captured during the weekly sampling strategy. Furthermore, our sampling strategy was constrained by adhering to a fixed sample size per grade, which may have contributed to lower detections of RSV.”
33. The observation that large class size is protective against viral infection is interesting. Could the authors indicate which age groups were streamlined into smaller or larger classes? Age group may be a significant confounder to the authors’ assertion that small class size may be protective.
34. The short paragraph on the study strength seems out of place in its current position: suggest relocate it to the end of the “limitations” paragraph, and reword as: “Nonetheless, our study has important strengths, including that symptoms data were collected, longitudinal testing was conducted, and a sensitive multiplex real-time assay was used to detect the targeted viruses.”
35. Please include the “n” and “N” in the sentence starting with “Second, we had many students…” in the paragraph which highlights the study limitations in the Discussion section.
36. In the sentence starting with “This could have biased…” in the paragraph on limitations in the Discussion section, suggest reword as: “This could have biased our estimation of viral infections circulating in the school to those associated with mild symptoms.”
37. In the third sentence of the last paragraph, suggest reword as: “… in school aged children…”
38. Please place the ‘question’ “Who infects whom?” in quotation marks.

Is the work clearly and accurately presented and does it cite the current literature?

Yes

If applicable, is the statistical analysis and its interpretation appropriate?

Yes

Are all the source data underlying the results available to ensure full reproducibility?

Yes

Is the study design appropriate and is the work technically sound?

Yes

Are the conclusions drawn adequately supported by the results?

Yes

Are sufficient details of methods and analysis provided to allow replication by others?

Yes

Reviewer Expertise:

Paediatric infectious diseases, particularly pneumonia.

I confirm that I have read this submission and believe that I have an appropriate level of expertise to confirm that it is of an acceptable scientific standard, however I have significant reservations, as outlined above.

# Response

**Published:** 2020-09-15

**Authors:** Irene Adema Wangwa

**Affiliations:** KEMRI Wellcome Trust Research programme, Kenya

**Responses to comments from  reviewer 1**

**Major Comment:**

**The authors mention that a limitation of their study was that they were unable to sample all symptomatic children in the study. This is important but needs to be elaborated on. What proportion of all symptomatic children in each grade were sampled? Were absenteeism lists maintained throughout the study to give an indication of the proportion of children that missed school days due to illness? Such information would assist in enabling a fuller understanding of the representativeness of the results achieved in this study**.

*During the study, all students in upper primary enrolled in the cohort were asked to self-report any ARI symptoms they experienced in a daily symptoms diary. Students in lower primary were monitored daily by study field staff and assisted in recording the ARI symptoms observed in their symptom diaries.  On the day of sample collection a* *count of all students presenting with ARI symptoms was first conducted from which the required target sample was selected to give a sample. The proportion of symptomatic students who were sampled in each grade has been included in the supplementary 1, proportions of symptomatic students sampled.*

*Records of absenteeism were captured in the symptom diaries used for symptomatic surveillance of ARI. Reasons for absenteeism were captured in the diaries.  More details of the causes of absenteeism are presented in a subsequent publication describing the symptomatic surveillance of symptomatic ARI in the school.*

**Minor comments**

*All minor comments have been addressed within the manuscript*.
